# Preface of Special Issue “21st Century Health Communication Challenges: Public Health Emergencies”

**DOI:** 10.3390/ejihpe13030042

**Published:** 2023-03-05

**Authors:** Carlos Ruíz-Núñez, Ivan Herrera-Peco

**Affiliations:** 1 PhD Program in Biomedicine, Translational Research and New Health Technologies, School of Medicine, University of Malaga, Blvr. Louis Pasteur, 29010 Málaga, Spain; 2 Faculty of Health Sciences, Universidad Alfonso X el Sabio, Avda. Universidad, 1, Villanueva de la Cañada, 28691 Madrid, Spain

Over recent years, the tendency to seek health information has increased exponentially worldwide. Although many scholars have linked this trend to global health emergencies, this is a pattern that has been observed before and may have been exacerbated by the COVID-19 pandemic [1]. The search for health information is becoming a daily concern for many people, many of whom suffer from pathologies about which they have insufficient information. This information deficit is associated with the emergence of negative emotions such as uncertainty, which can translate into stress, anxiety, frustration and even phobias [1,2,3].

The demand for information, combined with the large amount of available content, has resulted in infodemia, which represents a major health problem worldwide [4]. It is associated with increased uncertainty about what is and what is not evidence-based health information, leading to decreased adherence to health authority recommendations [5], but also making people less critical of the messages they receive and thus more likely to believe biased information [4,5]. When the need arises to consult doubts about a pathology or any health-related situation and access to a reliable source of information is not readily available, patients and their relatives seek information on the most convenient and accessible tool they have: the Internet [6].

Health disinformation was and continues to be a dangerous element for the health of individuals. However, it also jeopardizes the health care of the population and the management and sustainability of health systems. There are various actors involved in spreading erroneous, or confusing messages. These range from individual users, motivated by personal reasons, conspiratorial feelings [1,2,3], false beliefs that have been internalized [2,3], or simple confusion whereby misinformation is spread believing it to be true [6], to automated accounts or bots, identified as key elements in the dissemination of disinformation [3]. The latter actors have been especially important in efforts to discredit vaccination, nutrition, and cancer treatment or in the advocacy of miracle remedies. Although these accounts may represent a small quantity and proportion of users, they have a great capacity to influence conversations and, therefore, to modify the opinions of individuals.

It is in this context that healthcare professionals can play a leading role in generating and disseminating reliable information and can even contribute to the control of healthcare misinformation, thus helping to prevent its spread [3]. The public’s perception of these actors as reliable sources, coupled with their authority in health matters, represents a great opportunity. Amongst the primary resources at their disposal is scientific production via research publications in impact journals, and it is necessary to control the flow of cited articles. In this way, have an impact on avoiding the damage done to the body of knowledge when retracted literature is included in scientific manuscripts and can compromise the scientific reliability of knowledge in health sciences [6]. The involvement of health professionals requires institutional support in the form of specific training in scientific critical reading, research methodology, and communication.

In addition, it should be noted that, within the Internet, it is increasingly common to search for information through social media [1,3,4], which are consolidating as tools for the rapid and direct exchange of information. Those based on audiovisual content—such as Instagram, TikTok or YouTube—are becoming increasingly important, with written health content sometimes using language that is difficult to understand for people without adequate health literacy [3]. However, other social networks, such as Twitter and Facebook, continue to play an important role in the dissemination of health information [2,3].

This situation has triggered an important source of studies and research using social networks as primary sources. For example, one study [5] aimed at facilitating digital health research for public health professionals via the collection and analysis of Twitter conversations using generalized linear models (GLM) [5]. Research that can be followed up with unstructured data analysis to facilitate summaries of various information is of particularly high utility to advancing this research.

The Internet, along with the pandemic and its confinement, has also brought changes to formative methodology, affecting students. Indeed, it has been demonstrated [3] that the formative change proposed by online education had positive effects on academic performance, compared to the traditional face-to-face method. This has been particularly beneficial for younger students [3,7]. As has been observed, it has also given rise to [1] the justification for not using the masks, despite the recommendations, and the moral disconnection caused by dark personality traits, the so-called dark triad, as a self-regulatory mechanism to exclude moral self-censorship and justify ignoring recommendations to use the mask [1]. Despite the fact that current medical students are digital natives, there is still a need to improve their competencies in information and communication technology, enhance their skills and reinforce confidence to use eHealth information [7]. It is necessary for this reason to introduce literacy concepts into medical pedagogy along with workshops to improve evidence-based search and evaluation techniques to in order to acquire future competencies to foster health literacy.

Thus, it is necessary to increase the quantity of research being conducted that seeks to promote health, such as research that relates different socioeconomic variables and health in order to explain conditioning factors in this, as studied by [2]. In response to these challenges, we must propose public health intervention that improves the quality of life and reduces socioeconomic inequalities as a means of improving the quality of sleep [5,7].
